# *Synechocystis* sp. PCC 6803 shows high cell cycle dynamics reflected by an extraordinary genome copy number variation

**DOI:** 10.1186/s12934-026-02982-3

**Published:** 2026-04-27

**Authors:** Jacky Till, Juan López-Gálvez, Florian Schattenberg, Matthias Schmidt, Susann Müller, Jörg Toepel, Bruno Bühler

**Affiliations:** 1 Department of Microbial Biotechnology, Helmholtz Centre for Environmental Research – UFZ, Permoserstr. 15, 04318 Leipzig, Germany; 2 Institute of Chemical, Environmental and Bioscience Engineering, TU Wien, Gumpendorfer Str. 1a, Vienna, 1060 Austria; 3 Department of Applied Microbial Ecology, Helmholtz Centre for Environmental Research – UFZ, Permoserstr. 15, 04318 Leipzig, Germany; 4 Department of Technical Biogeochemistry, Helmholtz Centre for Environmental Research – UFZ, Permoserstr. 15, 04318 Leipzig, Germany; 5 Faculty of Natural Sciences I – Biosciences, Martin-Luther-University Halle-Wittenberg, Kurt-Mothes-Str. 3, 06120 Halle (Saale), Germany

**Keywords:** Cyanobacteria, Ploidy, Genome copy numbers, Flow cytometry, Phosphate starvation

## Abstract

**Background:**

Several cyanobacterial strains are known to contain a high number of genome copies and elevated cell cycle dynamics. We systematically investigated the ploidy of the model cyanobacterium *Synechocystis* sp. PCC 6803 during growth under conditions differing in type (light, CO_2_, phosphate) and extent of growth limitation.

**Results:**

The results obtained via flow cytometry with DAPI-stained cells and quantitative PCR revealed a direct correlation between growth rate and genome copy number (GCN), shedding light on the widely varying copy numbers reported in previous studies. The highest GCNs of up to 72 in average and well above 100 for individual cells were detected during unlimited growth. The lowest GCNs were measured upon limitation by CO_2_, light, and/or phosphate. Prolonged limitation by multiple factors led to the development of a small population with a GCN of 1 prone to facilitate genetic engineering. Additionally, under phosphate limitation, we found the development of distinct subpopulations differing in cell size, cell shape, as well as pigment and storage compound content, indicating a type of differentiation to survive stress conditions.

**Conclusions:**

This study deciphers the high GCN dynamics in *Synechocystis* sp. PCC 6803, thereby provides knowledge useful to determine the growth state of cells and for strain engineering (transformation, pathway engineering, etc.), and gives novel insights into physiological adaptations of cyanobacteria upon variation of environmental conditions.

**Supplementary Information:**

The online version contains supplementary material available at 10.1186/s12934-026-02982-3.

## Background

Cyanobacteria are an ancient and phylogenetically diverse group of Gram-negative bacteria that are capable to use light as energy source via oxygenic photosynthesis. In the last years, the interest in cyanobacteria has steadily increased, as they are considered aspiring candidates to function as photosynthetic cell factories in biotechnological applications [1–3]. However, the lack of genetic tools, the incomplete knowledge on their metabolism and its regulation, and intrinsic properties like their polyploidy limit their applicability today. Several cyanobacteria feature oligo- or polyploidy, which means that they contain up to 9 or more genome copies [4–8]. This also includes the model cyanobacterium *Synechocystis* sp. PCC 6803 (hereafter *Synechocystis*) [5, 8]. Oligo- or polyploidy is a common feature not only of cyanobacteria, but also of several heterotrophic bacteria and archaea [9–11]. Potential evolutionary advantages arising from the maintenance of multiple genome copies in prokaryotes have been discussed [9, 12, 13]. One key benefit is that additional chromosome copies can serve as a backup to cope with DNA damage caused by harsh environmental conditions. In cyanobacteria, this aspect is of importance, as their phototrophic lifestyle steadily exposes them to UV light and oxidative stress [14]. From the biotechnological point of view, ploidy variation is critical, as it affects physiological behavior and the introduction and maintenance of genes of interest, for which full segregation is necessary but often difficult to achieve. For the model organism *Synechocystis*, the genome copy number (GCN) is reported to vary in dependence of growth phase and environmental factors [5, 8]. In most studies, GCNs of 10–20 were reported in early growth phases [8, 15, 16]. Environmental factors reported to influence the GCN include light intensity as well as CO_2_ and phosphorus availability. An enhancing effect on the GCN was observed at low compared to high light intensities [8] and at increased CO_2_ levels (i.e., 2%) [15]. However, both factors have not been analyzed in more detail, and different studies diverge regarding absolute copy numbers as well as the effect of environmental conditions. The phosphorus concentration has been proposed to be the most important environmental factor influencing the GCN in *Synechocystis*. Prolonged cultivation under P-limiting conditions has been shown to drastically reduce the GCN [8, 17]. Zerulla and co-workers even observed a monoploid status after cultivation for 16 days in P-free medium. A similar effect of P-limitation has also been reported for other cyanobacterial strains such as *Synechococcus elongatus* PCC 7942 [18].

Beside environmental factors, a GCN change upon growth progression has been reported [5, 8, 17]. In batch cultures, cyanobacteria, after a lag phase, show exponential growth at µ_max_ until CO_2_ or light becomes limiting. For this phase, reported GCNs typically are in the range of 10–20 [8, 15, 16]. The highest reported GCN during exponential growth however amounts to 218 as stated by Griese and co-workers [5]. Later, the same group compared the number of chromosome copies in different strains and obtained much lower GCNs under the same conditions [8]. When light becomes limiting due to the increasing cell density, cyanobacterial cultures switch to linear growth, before growth stalls in a stationary phase. During linear or stationary phases, lower GCNs have been reported compared to the exponential phase [5, 8, 17], although the reported enhancing effect of low compared to high light intensities [8] appears to contradict this and, overall, reported maximum GCNs heavily differ. Further, the GCN typically has been determined for single or a limited number of selected time points as representatives for the respective growth phases, or the growth phase has not been considered at all. GCN dynamics during growth and respective effects of environmental factors have not been systematically analyzed up to date.

To close this knowledge gap and resolve divergences in literature regarding maximum GCNs as well as respective influencing factors and dynamics, this study aimed at a comprehensive analysis of GCN variation (i) in all growth stages and (ii) upon variation of multiple environmental factors. For this purpose, GCNs were comparatively determined via flow cytometry enabling single cell resolution and by qPCR as average in a culture. As environmental factors, we varied light intensity as well as CO_2_ and phosphorus availability. Accompanying physiology analyses included cell size, cell density, and growth rate. Thereby, this study provides a comprehensive picture regarding absolute GCNs and respective cell cycle dynamics in *Synechocystis*.

## Methods

### Strains and growth conditions

*Synechocystis* sp. PCC 6803 [19] was grown photoautotrophically in liquid yBG11 medium [20] buffered with 50 mM HEPES at pH 7.2, with phosphate concentrations of 175 µM (high phosphate, HP) or 17.5 µM (low phosphate, LP). Cultivation was performed in 100 ml, 250 ml, or 1 L baffled shaking flasks with cotton plugs at 30 °C, light intensities of 50 or 200 µmol photons m^− 2^ s^− 1^, ambient (0.04%) or 2% CO_2_, 75% humidity, and 150 rpm. Cell growth was monitored by measuring OD_750_ and analysis in a Multi-sizer™ 3 Coulter Counter^®^ (Beckman Coulter Life Sciences, Indianapolis, IA, USA). Growth on BG11 agar plates was conducted at 30 °C, 50 µmol photons m^− 2^ s^− 1^, ambient (0.04%) CO_2_, and 75% humidity.

### Determination of colony forming units (cfu)

10^6^ sorted cells were pelleted by centrifugation (10 min, 3,200 g, 4°C) and resuspended in 200 µl yBG11 medium. Dilution series were prepared and 10^6^, 7.5 × 10^5^, 10^5^, 10^4^, 10^3^, 5 × 10^1^, and 10^1^ cells contained in 200 µl cell suspension were plated on BG11 agar plates. Analysis of the dilution series was performed in technical duplicates. The plates were incubated at 30 °C and 50 µmol photons m^− 2^ s^− 1^ for 7 d. Colonies on plates of these dilutions were quantified as colony forming units (cfu), calculated as cfu = counted colonies / plated cells. Specified standard deviations are calculated from technical replicates of evaluable dilution plates.

### Time course experiments

Precultures for time course experiments were inoculated with cells from BG11 agar plates in 20 ml yBG11 medium in 100 ml flasks and cultivated at 30 °C, 50 µmol photons m^− 2^ s^− 1^, ambient CO_2_, and 150 rpm up to an OD_750_ of approximately 4. Time course experiments were performed under 8 different growth conditions varying three parameters, i.e., low and high light intensity (50 and 200 µmol photons m^− 2^ s^− 1^, LL and HL), low and high CO_2_ concentration (ambient CO_2_ and 2% CO_2_, LC and HC), and high and low phosphate concentration (yBG11 medium containing the usual high 175 µM and low 17.5 µM phosphate, HP and LP), respectively. This resulted in the following 8 conditions: LL-LC-HP, HL-LC-HP, LL-HC-HP, HL-HC-HP, LL-LC-LP, HL-LC-LP, LL-HC-LP, HL-HC-LP. Main cultures for time course experiments were inoculated from precultures to an OD_750_ of 0.05. For the inoculation of LP cultures, the cells were pelleted by centrifugation (5 min, 10,000 g, 4 °C), washed with 2 ml phosphate-free yBG11, pelleted again, and finally resuspended in yBG11 medium containing 17.5 µM phosphate. Duplicates of 200 ml main cultures where cultivated in 1 L baffled shaking flasks. Alternatively, 50 ml main cultures were cultivated in 250 ml baffled shaking flasks.

HL-HC (HP and LP) experiments were carried out for 7 days in baffled 1 L flasks. OD_750_ was measured for the preculture (inoculum) as well as at 0.5, 1, 1.5, 2, 2.5, 3, 4, 5, 6, and 7 d after inoculation. Flow cytometric analyses were prepared from the inoculum and at the same time points (plus 8 and 30 h) after inoculation. For qPCR analyses, samples for DNA extraction were prepared and cell concentrations determined using a Multi-sizer™ 3 Coulter Counter^®^ (Beckman Coulter) 1, 2, 3, and 7 d after inoculation.

LL-HC (HP and LP) experiments were carried out for 14 days in 1 L baffled flasks. OD_750_ was measured for the inoculum as well as 0.5, 1, 1.5, 2, 2.5, 3, 4, 5, 6, 7, 8, 9, 10, 12, and 14 d after inoculation. Samples for flow cytometric analyses were prepared from the inoculum and at the same time points (plus 8 and 30 h, but not 8, 9, and 12 d) after inoculation.

HL-LC (HP and LP) experiments were carried out for 10 days in baffled 1 L flasks. OD_750_ was measured for the inoculum as well as 0.5, 1, 1.5, 2, 2.5, 3, 4, 5, 6, 7, 8, and 9 d after inoculation. Samples for flow cytometric analyses were prepared from the inoculum and at the same time points (plus 8 and 30 h) after inoculation.

LL-LC (HP and LP) experiments were carried out for 35 d. OD_750_ was measured for the inoculum as well as 0.5, 1, 1.5, 2, 2.5, 3, 4, 5, 6, 7, 8, 9, 10, 12, 14, 17, 21, 24, 28, and 35 d after inoculation. Samples for flow cytometric analyses were prepared from the inoculum and at the same time points (plus 8 and 30 h) after inoculation.

HL-LC (HP and LP) (250 ml) and LL-LC-HP/LP (250 ml) experiments were carried out for 21 d. OD_750_ was measured for the inoculum as well as 1, 1.5, 2, 3, 4, 6, 7, 8, 10, 14, and 21 d after inoculation. Samples for flow cytometric analyses were prepared from the inoculum and at the same time points (plus 8 and 12 h) after inoculation.

### DNA extraction

For the extraction of genomic DNA (gDNA) from *Synechocystis*, 10^7^-10^8^ cells were harvested by centrifugation (10,000 g, 5 min, 4 °C), and the cell pellets were stored at -20 °C until further processing. DNA isolation was performed according to a universal extraction protocol [21] with slight modifications as described by Lettau and Till [22]. Final gDNA samples were resuspended in 30 µl ddH_2_O, and DNA quality and quantity were analyzed via Nanodrop One^C^ Spectrophotometer (Thermo Fisher Scientific, Waltham, MA, USA).

### Quantitative PCR (qPCR) for ploidy determination

Determination of the genome copy number (GCN) via qPCR was performed essentially as described by Griese and co-workers [5] based on a protocol originally developed for haloarchaea [11]. At first, a DNA standard fragment was generated from *Synechocystis* chr. 1107–2191 (same locus as in previous reports [5, 8, 11]). The 1085 bp DNA fragment was PCR-amplified from genomic DNA of *Synechocystis* using the primers qPCR_Syn_S1_for (CGCGGATTACCCTACCAGACC) and qPCR_Syn_S1_rev (GGTCCCAATACGGTTGGTAAGC) and Dream Taq polymerase (Thermo Fisher Scientific) according to manufacturer’s instructions. The PCR product was purified using “Nucleo Spin Gel and PCR-Cleanup KIT” (Machery Nagel, Düren, Germany) according to manufacturer’s instructions and eluted with 40 µl ddH_2_O. The quality and quantity of the standard DNA was analyzed via Nanodrop One^C^ Spectrophotometer (Thermo Fisher Scientific), and the DNA concentration was calculated based on the molecular weight computed with Geneious 10.2.6 using an online tool (http://www.molbiol.ru/ger/scripts/01_07.html). Dilutions containing 10^9^-10^2^ molecules DNA standard fragment / µl were prepared from a stock solution containing 10^10^ molecules/µl and used as DNA standards for qPCR.

qPCR analysis was performed in a StepOnePlus™ Real-Time PCR System (Applied Biosystems | Thermo Fisher Scientific) using PowerUp™ SYBR™ Green Master Mix (Applied Biosystems | Thermo Fisher Scientific) according to manufacturer’s instructions with slight modifications. The 10 µl reaction mix (prepared as a master mix) was set up on ice as follows: 5 µl PowerUp™ SYBR™ Green Master Mix + 0.45 µl of each primer (10 µM) + 3.1 µl ddH_2_O + 1 µl template. The primers qPCR_Syn_A1_for (CTAATCGCTGTGGTAGCCAAC) and qPCR_Syn_A1_rev (CAAGCCAGTGATGAGGTTAAAGC) were applied, yielding a 234 bp nested PCR product (chr. 1579–1819). DNA-standards (10^9^-10^2^ molecules/µl) and different dilutions of genomic DNA samples obtained from the time course experiments (1:10, 1:100, 1:1000 and 1:10,000) were measured in parallel. Analysis was carried out in technical triplicates. The following PCR protocol was run: 10 min initial denaturation at 95 °C, followed by 40 cycles of 15 s at 95 °C and 1 min at 60 °C. Threshold cycle (Ct) values were determined by StepOne Plus Software v2.3. Exponential regression of the PCR was verified by comparison of the Ct differences of the different dilutions (i.e., a 10-fold dilution corresponds to a Ct difference of about 3.32). A standard curve was generated and used to calculate GCNs for the analyzed DNA samples. Division by the number of cells (obtained via Coulter Counter analysis) finally gave an estimation of GCNs per cell. Mean values and standard deviations (SDEV) were calculated from technical triplicates and different dilutions each from biological duplicates. Statistical analysis was performed using SPSS. Analysis of variances (Welch-ANOVA) followed by a Games-Howell multiple comparison test was performed considering significance at *p* ≤ 0.05.

### Determination of GCN per single cell via flow cytometry

For time course experiments in 1 L flasks, cells from 2 ml liquid culture were pelleted by centrifugation (10 min, 3,200 g, 4 °C) and resuspended in the same volume phosphate buffered saline (PBS: 6 mM Na_2_HPO_4_, 1.8 mM NaH_2_PO_4_, 145 mM NaCl, pH 7) containing 2% (v/v) paraformaldehyde (PFA). After stabilization at room temperature (RT) for 30 min, cells were pelleted again by centrifugation (10 min, 3,200 g, 4 °C). The supernatant was discarded and the pellet briefly vortexed for 1 min, resuspended in 2 ml 70% EtOH, and stored at -20 °C. For time course experiments in 250 ml flasks, cells from 1 ml liquid culture were pelleted, stabilized in 1 ml 2% (v/v) PFA in PBS and finally resuspended in 1 ml 70% EtOH as described above.

Cells were stained with the highly AT-specific DNA dye 4’,6-diamidino-2-phenylindole (DAPI) to quantitatively highlight chromosome numbers per cell, as described [23]. In short, fixed cells were washed with PBS, and the cell solutions were adjusted to an OD of 0.035 (d_ʎ700nm_ = 5 mm) with PBS. Two ml of this solution were centrifuged again and the pellet resuspended in 1 ml of permeabilization buffer (0.11 M citric acid and 4.1 mM Tween 20 in bi-distilled water) and incubated at room temperature (RT) for 20 min. After another centrifugation step, the cells were stained with 0.24 µM DAPI in phosphate buffer (289 mM Na_2_HPO_4_ and 128 mM NaH_2_PO_4_ in bi-distilled water) overnight at RT. Before measurement, 0.5 and 1.0 μm UV Fluoresbrite microspheres (Polysciences, Warrington, PA, USA) were added to the stained cells as an internal marker for measurement accuracy.

The cells were measured using a BD Influx v7 Sorter with the BD FACS Sortware 1.2.0.142 (Becton, Dickinson and Company, Franklin Lakes, NJ, USA). The instrument was equipped with a 488 nm Sapphire OPS laser (400 mW) and a 355 nm Genesis CX laser (100 mW, both Coherent, Santa Clara, CA, USA). The 488 nm laser light was used to detect the forward scatter (FSC, 488/10 nm band pass filter, PMT 1) related to cell size, the side scatter (SSC, 488/10 nm band pass filter, PMT 2 trigger signal) related to cell density, and the autofluorescence of phycobilins (616/23 nm band pass filter, PMT 5). The DAPI fluorescence (460/50 nm band pass filter, PMT 9) related to cellular chromosome numbers was measured using the 355 nm laser. Light was detected by Hamamatsu R3896 PMTs in C6270 sockets (Hamamatsu, 211 Hamamatsu City, Japan).

The fluidic system was run at 33 psi sheath pressure using a 70 μm nozzle. As sheath fluid, FACSFlow buffer (BD) was used. Samples were measured and sorted at a speed of around 3000 events s^− 1^. For the daily optical calibration of the cytometer in the linear range, 1 μm bluefluorescent FluoSpheres (Molecular Probes, F-8815, Eugene, OR, USA) and 2 μm yellow-green fluorescent FluoSpheres (ThermoFisher Scientific, F8827, Waltham, MA, USA) were used. For calibration in the logarithmic range, 0.5 and 1 μm UV Fluoresbrite Microspheres (Polysciences) were applied. In addition, a mock community consisting of 3 strains was stained every measurement/sorting day (same as the samples described above) as a biological staining and quality control (as described [23]). Before measurement, the stained samples were filtered with a 50 μm mesh filter CellTrics (Sysmex Partec) to prevent clogging of the nozzle. All cytometric raw data can be accessed at Zenodo (https://zenodo.org/records/17241600?*).*

**Fig. 1:**
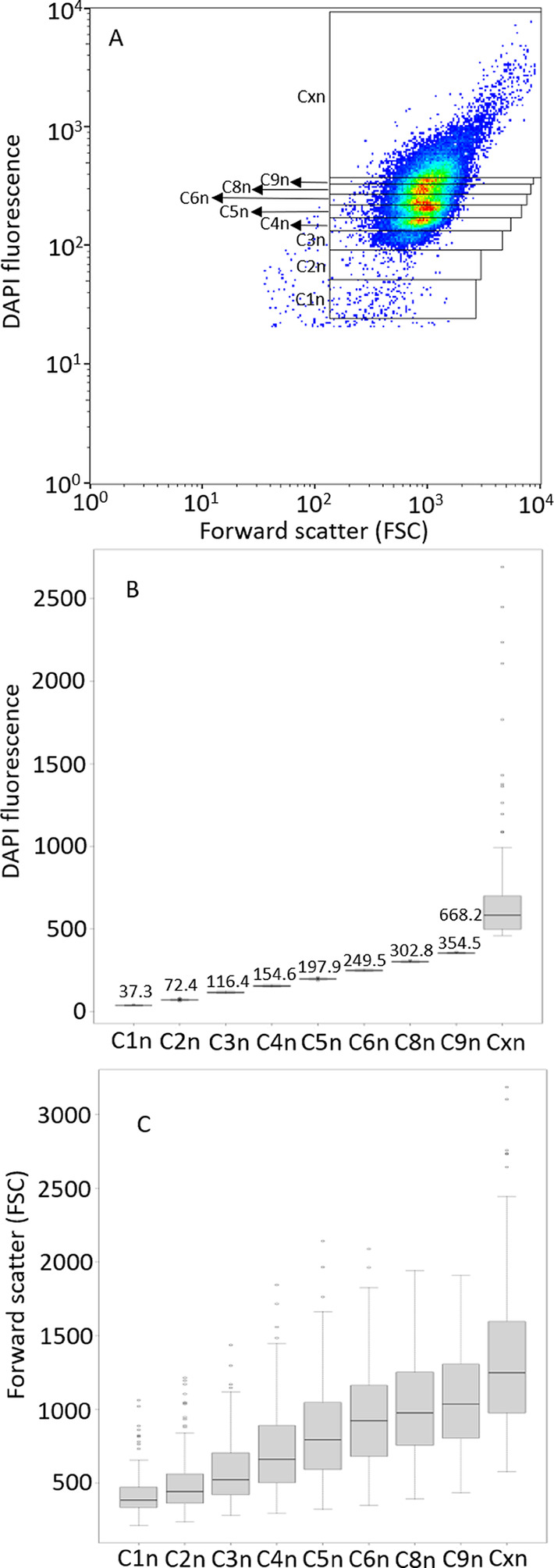
Distinct subpopulations differing in GCN as detected via flow cytometric analyses of *Synechocystis* cells. **A** Exemplary 2D-plot of a typical FSC vs. DAPI distribution of *Synechocystis* cells sampled from a HL-HC-HP-1 L culture at day 7. Each dot represents a cell, and the color changes from blue to red refer to increasing numbers of cells per position in the 2D plot. The overlay represents the gate template applied to count proportions of cells in subpopulations according to their DAPI fluorescence intensity. The gates are defined according to the Gaussian distribution of cells with corresponding chromosome numbers and are designated as C1n for 1 GCN per cell to Cxn for more than 9 GCNs per cell. This gate template was used for all flow cytometric analyses performed in the study. **B** and **C** show the mean DAPI fluorescence intensities and FSC values, respectively, for each gate (C1n to Cxn) and were derived from data of all flow cytometric analyses performed in the study including respective standard deviations

After generating a cell gate to exclude beads and noise in a 2D plot forward scatter (FSC) against DAPI fluorescence (50.000 cells per sample), 10 gates were defined to create the gate template to mark all subpopulations in all measured samples (see Fig. 1). The gates (G) determine the number of chromosomes per cell using the FI intensity of DAPI (y-axis, log scale) and thus mirror the GCN. Cells with identical GCN are visualized as separate gates, named C1n to Cxn, with C1n being the lowest GCN observed in this study. Cxn is the highest GCN, which is the sum of all cells that could not be assigned to a specific lower number of chromosomes. To determine cell numbers per gate for C1n to Cxn, the FlowJo version v10.8.1 software was used (FlowJo LLC, Oregon, USA).

### Cell sorting

For generation of the two cell subpopulations of different size, *Synechocystis* was grown in 50 ml yBG11 medium containing 17.5 µM phosphate at 30 °C, 200 µmol photons m^− 2^ s^− 1^, 2% CO_2_, 75% humidity, and 150 rpm for 7 days. 10^7^ cells of each of the two subpopulations were live cell sorted using the most accurate sort mode “1.0 drop Pure” and 4-way sorting according to autofluorescence (616/23 [488]) and FSC (correlating to the cell size). Per sample, 2 × 10^6^ cells were sorted 5 times for microscopy.

### Helium ion microscopy (HIM)

10^7^ sorted cells or cells directly from the culture were pelleted by centrifugation (10 min, 3,200 g, 4 °C) and fixed in 2 ml 0.8% (v/v) glutaraldehyde in 0.2 M sodium cacodylate buffer, pH 7.4, at 4 °C o/n. The suspension was then slowly filtered through a 25 mm polycarbonate filter paper with a pore size of 0.22 μm (Merck-Millipore, Burlington, MA, USA) using a manual filtration unit (Sartorius, Göttingen, Germany). In preparation for HIM, the filter had been sputter-coated with a 30 nm thick gold-palladium layer before filtration in order to avoid electrical charging under the ion-beam. Thereafter, the filter papers with the fixed cells on them were removed from the filtration device and rinsed twice with sodium cacodylate buffer for 5 min. Subsequently, the samples were dehydrated in a graded aqueous ethanol series (30, 50, 70, 80, 90, 95, and 100% EtOH) by incubation for 2 min each. The samples were then first stepwise shifted to hexamethyldisilazane (HMDS) by incubation in a 1:1 ratio mix of HMDS and EtOH and then in 100% HMDS for 10 min each. Finally, the filter papers with the dried sample were air dried under a fume-hood. High-resolution scanning helium ion microscopy was performed with a Zeiss Orion NanoFab (Carl Zeiss Microscopy, Peabody, MA, USA), using an ion-landing-energy of 25 keV and a beam current of 0.2–0.8 pA. For imaging, secondary electrons were detected with an Everhart-Thornley type detector. Before the experiments, the lateral resolution of the beam was checked and found to be typically better than 3 nm. In some cases, such as the imaging of larger *Synechocystis* cells at high magnification, the electron flood-gun of the HIM was used to reduce charging of the cells, resulting in darkening and low contrast.

### Scanning electron microscopy in transmission mode (STEM)

10^7^ sorted cells or cells directly from the culture were pelleted and fixed as described above for HIM. After fixation, the cells were centrifuged (10 min, 3,200 g, 4 °C), washed in 1.5 ml sodium cacodylate buffer, transferred to a 1.5 ml Eppendorf tube and centrifuged again. The obtained pellet was resuspended in 30 µl sodium cacodylate buffer containing 4% agar (pre-melted on a thermoblock at 95 °C for 30 min) and kept at RT, until the agar solidified. The piece of agar was detached from the wall of the Eppendorf tube, and the immobilized cells were post-fixed and stained with 1% osmium tetroxide solution (Electron Microscopy Sciences, Hartfield, PA, USA) in sodium cacodylate buffer for 30 min at RT. Next, the cells were partially dehydrated in a graded aqueous ethanol series (10, 30, and 50% EtOH) by incubation for 30 min each, followed by staining with 1% uranyl acetate in 70% EtOH for 1 h. After incubation in 70% EtOH at 4 °C o/n, the cells were completely dehydrated by incubation in 70, 90, and twice in 100% EtOH for 30 min each. Subsequently, the samples were stepwise shifted to LR white resin (Agar scientific, Stansted Essex, UK) by incubation in LR white-EtOH mixtures (LR white to EtOH ratios of 1:2 and 2:1) and two times in pure LR white for 30 min each. Finally, the cells were embedded in a resin. For this purpose, the piece of agar containing the cells was transferred into a nitrocellulose-fibrils capsule, covered with LR white, and kept in an oven at 60 °C until complete solidification of the LR white (3–4 days). The tip of the solidified resin block was trimmed to a flattened pyramid shape with a diamond knife using a trimmer from Leica microsystems (Wetzlar, Germany). The surface of the flattened pyramid tip was well polished, and ultra-thin slices (down to 70 nm in size) were cut with a Leica EM UC7 microtome (Leica Microsystems) using a glass knife. Scanning electron microscopy in a transmission mode (STEM) was performed with a Zeiss Merlin VP Compact (Carl Zeiss Microscopy, Oberkochen, Germany), using an electron acceleration voltage of 10 kV and a beam current of about 250 pA. The lateral resolution measured as edge contrast directly on the samples was about 4 nm.

## Results

### *Synechocystis* shows populations with distinct GCNs during photosynthetic growth

In order to investigate GCN dynamics in *Synechocystis* across different stages of growth under different environmental conditions, DNA contents were quantified during extended cultivation under conditions differing in light, CO_2_, and phosphorus availability. For cultivations in standard yBG11 medium and 1 L or 250 mL flasks (see Materials and Methods for details), light intensities of 200 and 50 µmol photons m^− 2^ s^− 1^ (high and low light; HL and LL), gaseous CO_2_ levels of 2% and ambient 0.04% (high and low carbon; HC and LC), and phosphate concentrations of 175 and 17.5 µM (high and low phosphate; HP and LP) were applied.

Figure 1A shows an exemplary cytometric 2D plot of DAPI-stained cells of *Synechocystis* (HL-HC-HP-1 L-7d) including the gate template used for the evaluation of all flow cytometric analyses. For DAPI fluorescence intensity (FI) values, discrete subpopulations could be distinguished that correspond to specific GCNs and allow the definition of gates labelled C1n (referring to one chromosome per cell) to C9n (referring to nine chromosomes per cell, Fig. 1A). C1n is the gate with the lowest GCN, while Cxn pools the cells with the highest GCNs. The variation in DAPI FI per cell for cells having different GCNs highlights replication and cell division activities (Fig. 1B). Cells belonging to gates C1n, C2n, C4n, and C8n represent replication followed by symmetric cell division. Respective mean DAPI FIs (in arbitrary units) are 37.3, 72.4, 154.6, and 302.8. Cells belonging to gates C3n, C5n, C6n, and C9n represent replication followed by asymmetric cell division with respective mean DAPI FIs of 116.4 (~ C1n + C2n), 197.9 (~ C1n + C4n), 249.5 (~ 2 x C3n), 354.5 (~ C4n + C5n). Whereas gate numbering followed this consideration and C1n FI multiplicability, other combinations are also possible. Cxn cells represent cells with higher GCNs or small cell aggregates. Cells in gate Cxn showed an average DAPI FI value of 668.2 and a high variation, as expected for this large gate including a wide range of GCNs. Cell-size-related FSC increased with the GCN (Fig. 1C). The average FSC signals ranged from 419 ± 144 in gate C1n to 1065 ± 366 in gate G9n and 1343 ± 498 in gate Cxn. While G9n shows a ~ 9-fold increase in GCN compared to C1n, the FSC increased only ~ 2.5-fold, which can be attributed to either an increase in cell size or a higher number of cells in the division state. The data for each gate were obtained as averages over all conditions and time points analyzed.

Thus, flow cytometry based on DAPI fluorescence allowed the clear differentiation of subpopulations with distinct GCNs and the setting of corresponding gates C1n to C9n following a well-established procedure [10]. Together with gate Cxn, summarizing the cells with GCNs above the one in C9n, this enabled the analysis of GCN dynamics during growth under different conditions as given in detail in the following sections.

### *Synechocystis* cells exhibit a highly dynamic GCN depending on growth state and light intensity

**Fig. 2:**
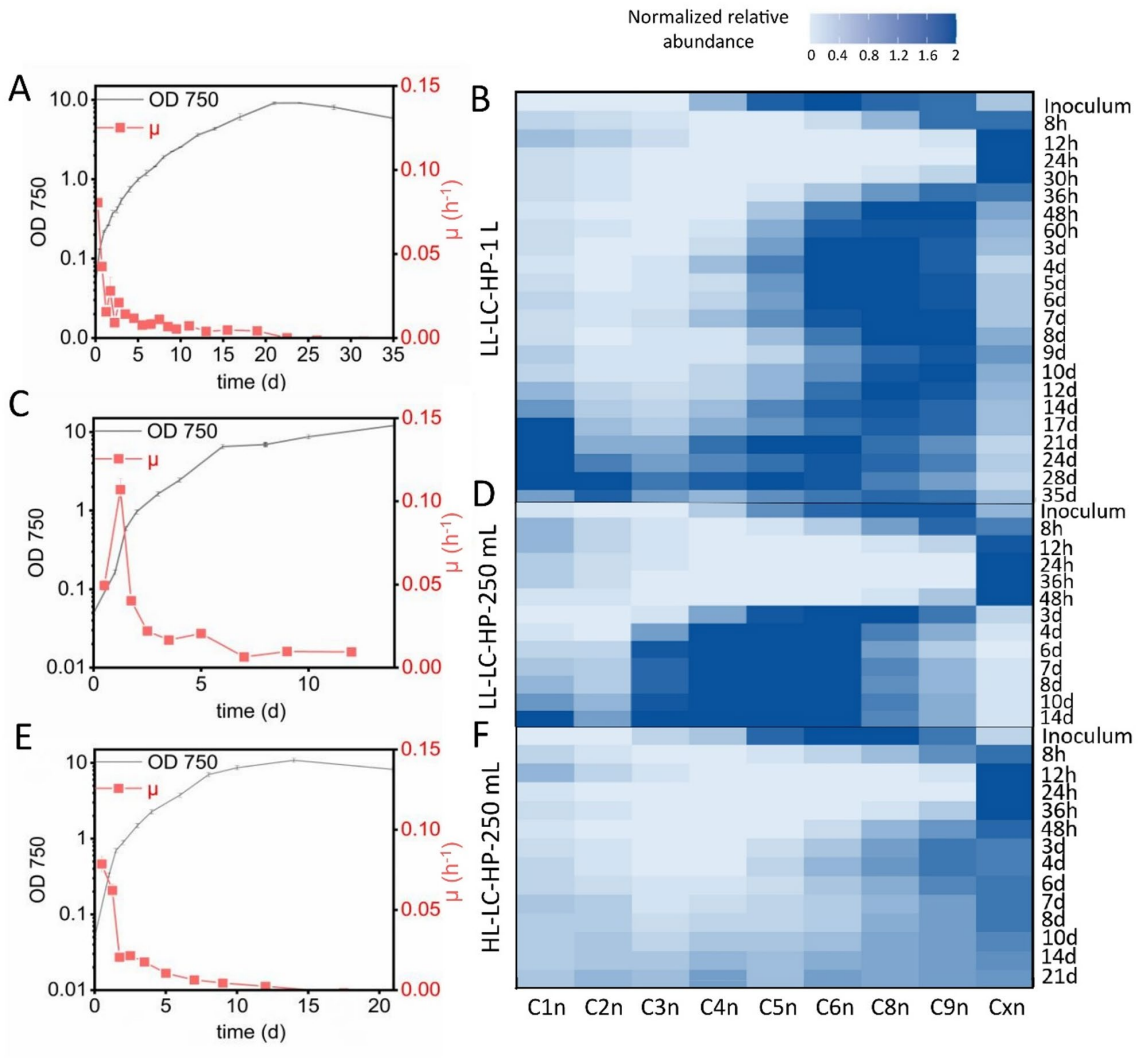
Growth- and time-dependent GCN dynamics in *Synechocystis* cultivated at low CO_2_ availability. Cultures grown at LL and HL conditions combined with LC and sufficient phosphate supply (LL-LC-HP, **A**–**D**, and HL-LC-HP, **E** and **F**) in 1 L (**A** and **B**) and 250 mL flasks (**C**–**F**) were analyzed. Growth curves (OD_750_) are plotted in (**A**, **C**, and **E**) for all conditions, together with µ courses based on values calculated between sampling time points. **B**, **D**, and **F** show GCN patterns over time analyzed by flow cytometry on the basis of DAPI fluorescence, with the cell signals assigned to defined gates (see Fig. 1 and Materials and Methods for experimental details). Darker colors represent higher relative cell numbers of cells per gate

The analysis of GCN dynamics in *Synechocystis* was started by cultivating cells at a low light intensity, ambient CO_2_, and high phosphorus levels in 1 L flasks containing 200 mL standard yBG11 medium (LL-LC-HP-1 L). Cells showed an initial growth rate of µ ≈ 0.08 h^− 1^, which first sharply decreased followed by slow growth (Fig. 2A). In order to reduce the presumably strong CO_2_ mass transfer limitation, this cultivation was also done in 250 ml flasks containing 50 mL medium, which resulted in a similar initial µ, which decreased more slowly in the first days (Fig. 2C). This led to higher maximum cell densities, which were achieved faster compared to cultivation in 1 L flasks. At high light intensity and otherwise the same conditions (HL-LC-HP), a similar growth behavior was observed in the two flask setups as under LL-LC-HP conditions (Fig. 2E, Fig. S1A) indicating that light was not a growth-limiting factor but CO_2_ limitation was dominant. In all cases, the GCN strongly increased during the initial growth phase followed by a decrease to GCNs as low as C1n at the end of cultivations. Under high light conditions, higher maximal average FIs per cell were observed in population Cxn compared to low light conditions (1196 compared to 863 and 2107 compared to 1086 arbitrary units in 1 L and 250 mL flasks, respectively, after 24 h of cultivation). Obviously, an increase in light availability, while not having a significant effect on CO_2_-limited growth, led to higher maximal GCNs.

LL-LC-HP cultures in 1 L flasks were analyzed for a longer term, i.e., 35 days. This long cultivation process revealed that, after the initial sharp increase in GCNs during fast growth, GCNs decreased and then remained relatively constant within the C6n to C8n range during slow growth until days 12–14 (Fig. 2B). Thereafter, the GCN further decreased with C1n becoming the dominant subpopulation. A similar behavior was observed for LL-LC-HP cultures in 250 mL flasks with C4n, C5n, and C6n as most populated gates during slow growth (Fig. 2D). However, under high light conditions, the decrease in GCNs was less pronounced, with many cells remaining in Cxn and becoming more evenly distributed among subpopulations. It can be suggested that cells may preserve more genome copies under these conditions (Fig. 2F, S1B).

Summed up, generally high GCNs and respective dynamics were observed under high phosphorus availability, especially during the first 2 days. An increase in light intensity under ambient CO_2_ conditions resulted in higher GCNs over all growth stages and a higher GCN variability among cells in late growth stages. Furthermore, partially relieving CO_2_ limitation by improving CO_2_ mass transfer slightly prolonged high-rate growth correlating with a prolonged maintenance of maximum GCNs in the early growth phase.

### During unlimited growth, *Synechocystis *cells reach extraordinarily high GCNs

To prove the promoting effect of higher CO_2_ availability on GCN dynamics, the impact of relieving the CO_2_ limitation on the GCN was investigated. For this purpose, cells were cultivated at high CO_2_ levels (2%, HC) again under LL and HL conditions (i.e., 50 and 200 µmol photons m^− 2^ s^− 1^, respectively) in 1 L flask setups (LL-HC-HP-1 L and HL-HC-HP-1 L, respectively).

**Fig. 3:**
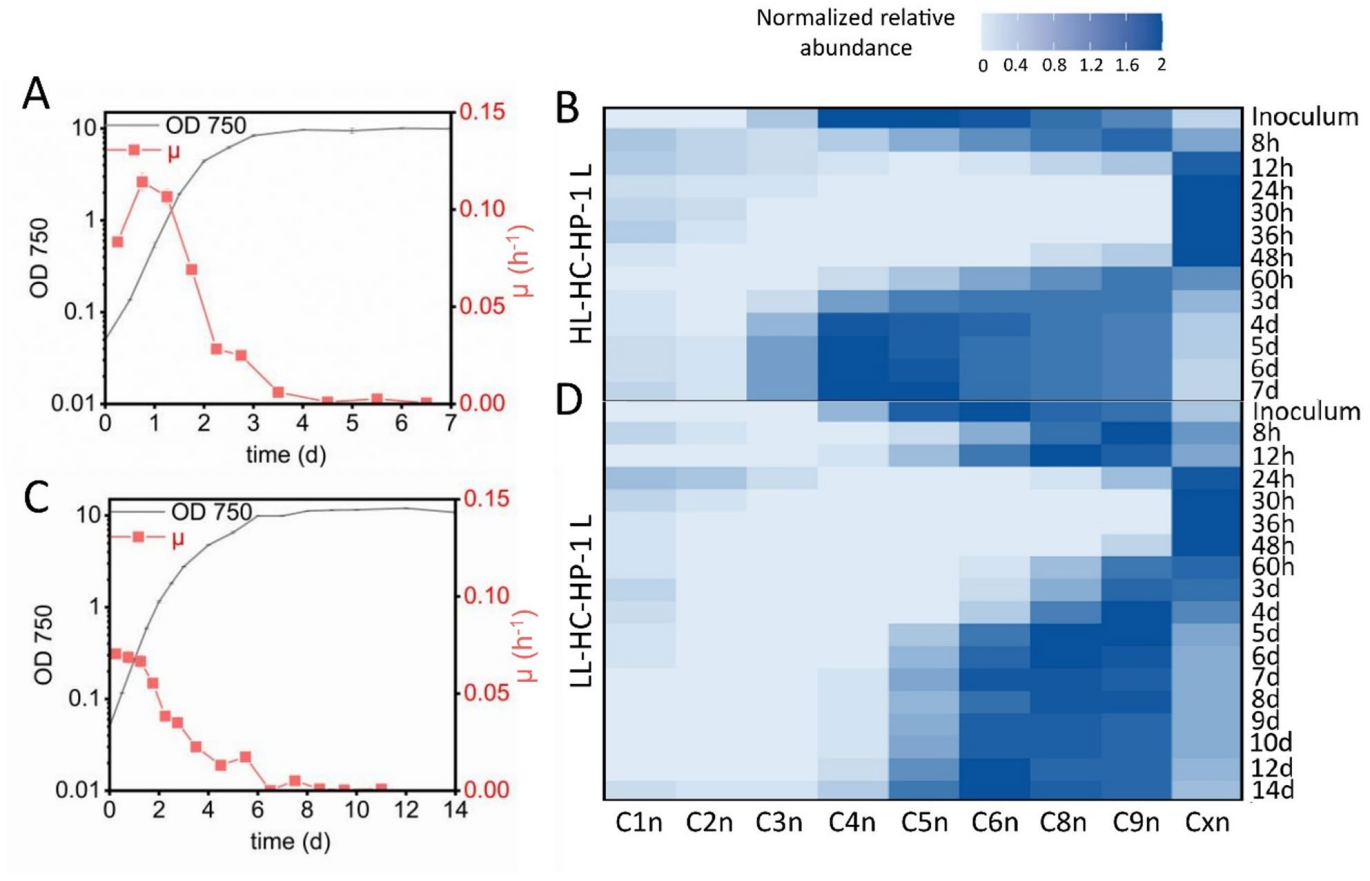
Growth- and time-dependent GCN dynamics in *Synechocystis* cultivated at high CO_2_ availability. Cultures grown under HL-HC-HP-1 L (**A** and **B**) and LL-HC-HP-1 L (panels C and D) conditions were analyzed. **A** and **C** show growth curves (OD_750_) and µ courses (µ calculated between individual sampling time points). **B** and **D** show GCN patterns over time under the two conditions analyzed by flow cytometry on the basis of DAPI fluorescence, with the cell signals assigned to defined gates. Darker colors represent higher relative cell numbers of cells per gate

As expected, growth was improved under HC conditions. In the initial phase, the maximal specific growth rate µ_max_ under HL-HC-HP conditions (~ 0.11 h^− 1^, Fig. 3A) clearly surpassed that achieved under HL-LC-HP conditions (~ 0.08 h^− 1^, Fig. 2A). Further, the exponential phase was prolonged until an OD_750_ of 2 was reached after 1.5 days, indicating unlimited growth in this phase, which was followed by linear growth and decreasing µ with light as limiting factor (Fig. 3A). Under LL-HC-HP conditions, unlimited growth was also maintained longer than at low CO_2_ availability, but the µ during this phase (~ 0.07 h^− 1^) was lower compared to HL-HC-HP conditions, indicating the expected light limitation effects (Fig. 3C).

Again, the GCN strongly increased in the initial growth phases (Fig. 3B, D). The prolongation of growth at high rates under HL-HC-HP conditions, with unlimited growth for 30–36 h, correlated with a prolonged increase and incidence of high GCNs. Up to 48 h, the majority of the cells accumulated in subpopulation Cxn (> 90% of the cells), reaching a maximum average fluorescence intensity value up to 2,690 [arb. FI units] at 30 h. Based on the mean fluorescence of 37.3 [arb. FI units] in C1n and assuming a doubling of GCNs from C1n to C2n (and then further to C4n and C8n), this value correlates with a ~ 72-fold increase in GCN. As light became limiting, the cells accumulated fewer genome copies, and the gates between C3n and C9n became the most populated ones after 4 to 7 days of cultivation. Cells cultivated under LL-HC-HP conditions demonstrated comparable behavior, yet they displayed a notably diminished maximum average fluorescence (701 [arb. FI units]) in Cxn at 30 h. Additionally, a less diverse distribution of GCNs was observed, with the GCNs mainly present in the C6n and C8n fractions between days 5 and 14 of cultivation. Thus, growth restriction via light limitation led to lower GCN maxima and dynamics. Unlimited growth on the other hand led to exceptionally high GCNs in *Synechocystis*, again indicating a strong correlation of the GCN course with growth phases and rates.

### GCNs in *Synechocystis *cells mainly correlate with the growth rate and are reduced by phosphate limitation

**Fig. 4:**
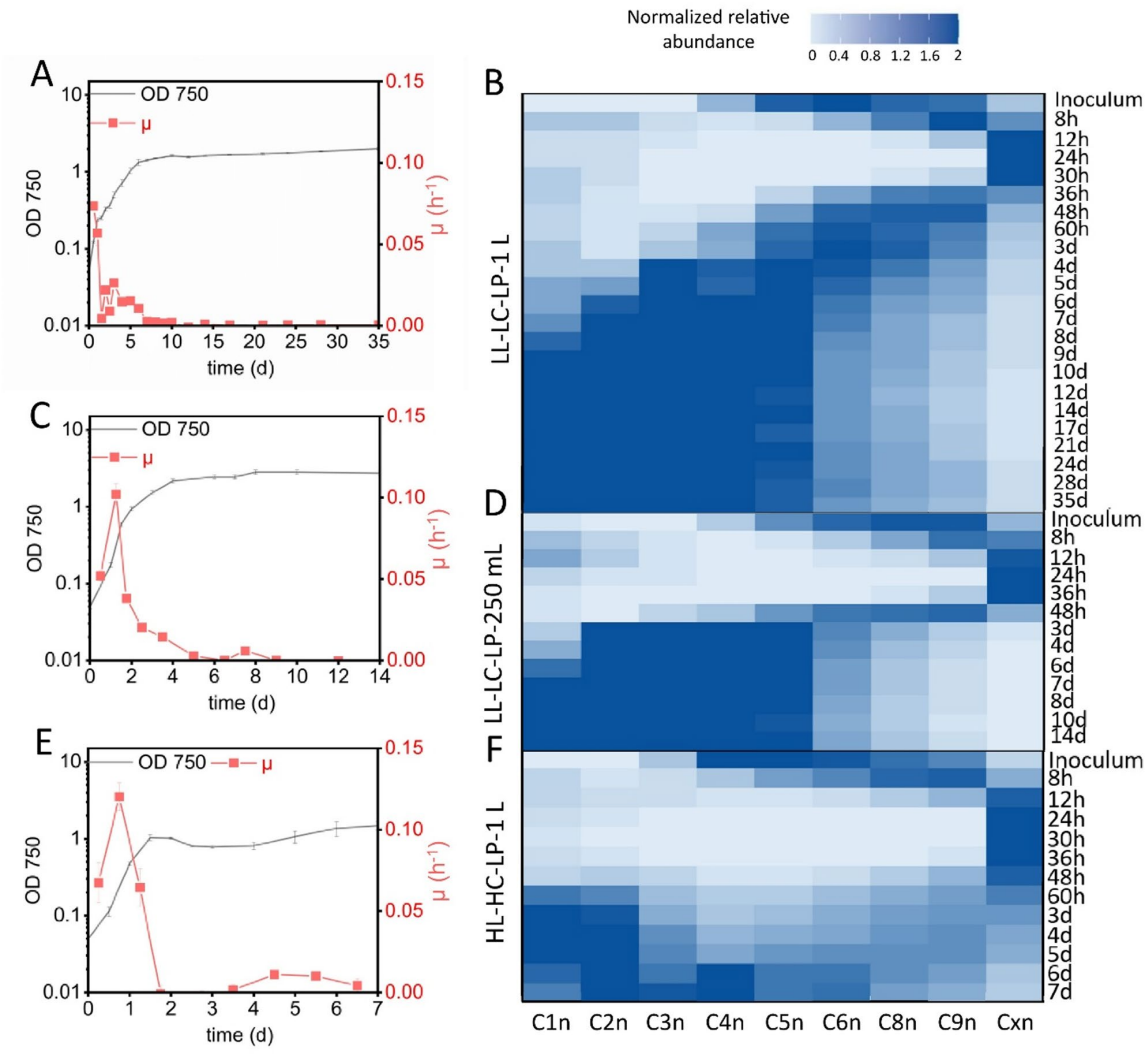
Growth and time-dependent GCN dynamics in *Synechocystis* cultivated under phosphorus-limited conditions. Cultures grown at LL-LC-LP conditions in 1 L (**A** and **B**) and 250 ml (**C** and **D**) flasks and under HL-HP-LP conditions in 1 L flasks (**E** and **F**) were analyzed. **A**, **C**, and **E** show growth curves (OD_750_) together with µ courses based on values calculated between sampling time points. **B**, **D**, and **F** show GCN developments over time analyzed by flow cytometry on the basis of DAPI fluorescence, with the cell signals assigned to defined gates. Darker colors represent higher relative cell numbers of cells per gate

Finally, low phosphate concentrations (LP) were tested for all conditions, a factor reported to influence GCNs of cyanobacteria [8, 17]. Whereas LP conditions generally resulted in similar initial growth rates as observed under HP conditions, the time span for growth at high rates was shorter, especially under HL conditions, and lower maximal OD values were obtained (Fig. 4A, C, E and Fig. S1C, E, G). This was consistent with an initially high proportion of cells in the Cxn subpopulation, whose maximum average fluorescence intensity was reached earlier than under HP conditions (Fig. 4B, D, F and Fig. S1D, F, H). Under HL-HC-LP for example, the maximum average fluorescence intensity (1375 [arb. FI units]) was reached after only 24 h instead of 30 h (Fig. 4F). For all applied conditions, the majority of cells subsequently shifted quickly and massively towards low GCN subpopulations, in most cases C1n-C5n (4BDF and Fig. S1D, F, H). No major differences were observed for cultivation in 250 ml and 1 L flasks under LL-LC-LP and HL-LC-LP conditions.

It can be concluded that the occurrence of P-limitation significantly affected DNA synthesis after 24–36 h and caused very low GCN numbers by the end of the cultivation period. High light conditions seem to slightly delay this effect (Fig. 4F, Fig. S1F, H). Overall, GCNs were found to correlate with µ under all conditions.

Moreover, an interesting phenomenon became visible in the late phase of cultivation under LP conditions. Two subpopulations of cells with different sizes emerged in flow cytometric analyses, as observed in 2D plots of FSC vs. DAPI fluorescence, which was further analyzed in Sect. “*Synechocystis* cells show distinct populations varying in cell size upon long term cultivation under LP conditions”.

### *Synechocystis* cells show very high GCNs and respective dynamics

**Fig. 5:**
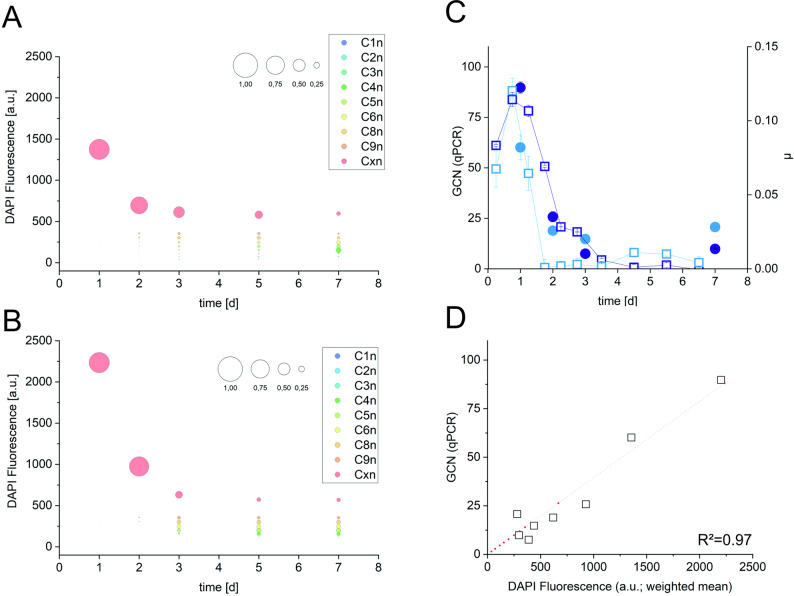
GCN dynamics of *Synechocystis* determined via quantitative PCR (qPCR) compared to flow cytometric analyses. The two methods correspond to population- and single-cell-level analyses, respectively. **A** and **B** show the proportional distributions of the mean DAPI fluorescence values in the different gates C1n-Cxn obtained from flow cytometric analyses of cultures grown in 1 L flasks under HL-HC-LP and HL-HC-HP conditions, respectively. The circle sizes represent the proportion of cells per gate. **C** shows courses of GCNs obtained from qPCR analysis (filled circles) and growth rates (open squares) for *Synechocystis* cells cultivated under the same conditions, HL-HC-HP (dark blue) and HL-HC-LP (light blue). qPCR analyses were performed 24 h, 48 h, 3 d, and 7 d after inoculation. **D** shows the correlation of qPCR data with weighted mean values of DAPI fluorescence over all gates (C1n-Cxn, empty squares) at corresponding time points, based on a linear regression. All experiments were carried out in duplicates (mean and standard deviation calculated accordingly)

DAPI binding to RNA is known not to interfere with DAPI-based GCN determination under the conditions applied [24], but interference of DAPI-stained plasmid DNA can occur, as *Synechocystis* sp. PCC 6803, with a genome size of 3.57 Mb, features 7 native plasmids with a cumulative size of ~ 383 kb and copy numbers in the range of 3–15 for the 4 larger plasmids (together ~ 373 kb) and 15–70 for the 3 small plasmids (together ~ 10 kb) [25]. To validate the GCN dynamics measured on a single cell level via DAPI staining and flow cytometric analyses and to get a quantitative insight into population-wide average GCNs, we thus performed quantitative PCR (qPCR) analyses based on a genomic sequence (see materials and methods for details) for the cultivations under HL-HC-HP and HL-HC-LP conditions (Fig. 5).

The GCN time courses determined via qPCR coincided with and confirmed the observations at the single cell level via flow cytometry. That is, high GCNs in the initial growth phases were followed by a decrease in GCNs with increasingly limiting resources and accordingly decreasing growth rates. Under HL-HC-LP and HL-HC-HP conditions, maximum GCNs of 60.1 +/- 6.2 and 89.8 +/-2.85 were determined via qPCR after 24 h, corresponding to maximal mean fluorescence values of 1375 and 2235 [arb. FI units] in Cxn (> 90% of the cells) measured at the same time points. GCNs estimated via qPCR generally correlated well with overall means of DAPI fluorescence as confirmed by linear regression (Fig. 5D), which indicated a GCN of 1 for C1n (1.45 based on the slope of 0.039 and the 37.3 [arb. FI units] of C1n, see Fig. 1B). Under HL-HC-HP conditions, the mean fluorescence in Cxn reached a maximum of 2,690 after 30 h, a 72-fold increase over that in C1n. Individual cells showed values in the range of 10,000 (Fig. S2) corresponding to a GCN as high as 268. These results infer that GCNs in *Synechocystis* can vary between 1 and well above 100.

The qPCR results confirm the results obtained via DAPI staining and flow cytometry and indicate that inaccuracies due to DAPI stained plasmid DNA were minor, but may explain the rather high copy number of 1.45 estimated for C1n based on its possibly plasmid-DNA-enhanced DAPI fluorescence correlated to GCNs determined by qPCR (Fig. 5D). Given the largely independent replication of plasmid- and genomic DNA [26], high GCNs determined via DAPI staining may have been slightly underestimated. Overall, it can be concluded that the GCN dynamics are influenced by cultivation conditions, such as light, CO_2_, and phosphorus availability. Importantly, growth behavior and dynamics resulting from changes in cultivation conditions and the named factors led to substantial GCN variation.

### *Synechocystis* cells show distinct populations varying in cell size upon long term cultivation under LP conditions

**Fig. 6:**
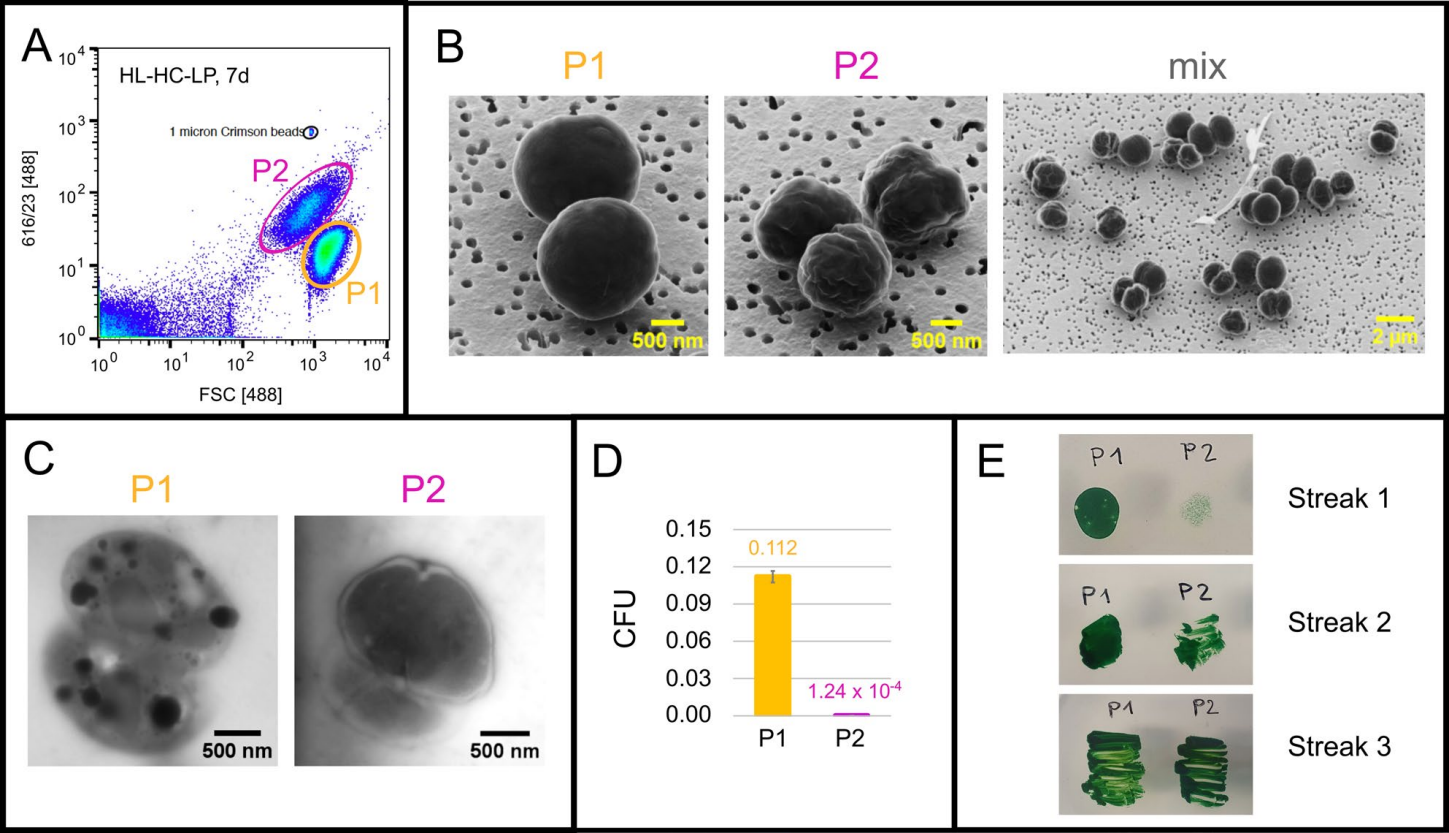
*Synechocystis* subpopulations with different cell size formed after growth under HL-HC-LP conditions for 7 d. Subpopulation differences in shape and contour were analyzed by flow cytometry (**A**), HIM (**B**), and STEM (**C**). Cultivability was tested by CFU determination (**D**) and re-cultivation on agar plates (**E**). Panel A shows a 2D dot plot of autofluorescence (excitation at 488 nm, emission detected at 616 nm +/- 23 nm) against the forward scatter (FSC) for PFA-fixed cells. Fresh cells were then re-cultivated and live-cell-sorted according to the indicated subpopulations P1 and P2 (see Fig. S3A). Panel B shows HIM images of sorted and unsorted cells (mix). Panel C shows STEM pictures of cells derived from the two subpopulations. Panel D shows CFU analysis performed for live-sorted cells from P1 and P2. Panel E shows the re-cultivation of cells from P1 and P2

In the late/stationary growth phases under all tested LP conditions, cells diverged into two subpopulations differing in cell size and autofluorescence at 616 +/- 23 nm upon excitation at 488 nm (Fig. 6A). We further analyzed these subpopulations via microscopy. To this end, live cells that had been re-cultivated in a time-course experiment under HL-HC-LP conditions for 7 d were analyzed again cytometrically, and the respective cells of subpopulations 1 (P1, large cells) and 2 (P2, small cells) were live-cell-sorted. Detected subpopulations essentially matched those detected after PFA-fixation demonstrating that fixation does not affect cell morphology (Fig. 6A, Fig. S3AB). For final analysis of subpopulations P1 and P2, 10^7^ live-cell-sorted cells were fixed with glutaraldehyde and prepared for helium ion microscopy (HIM) and scanning electron microscopy in a transmission mode (STEM). An unsorted sample was prepared as a control (Fig. 6B [mix]).

HIM analyses confirmed the difference in cell size between subpopulations P1 and P2 (Fig. 6B and Fig. S4A). Further it showed that cells from P1 were round and evenly shaped, whereas the smaller cells from P2 featured a wrinkled surface. The unsorted control sample contained both phenotypes. STEM analysis indicated that cells from P2 contained less granular inclusions than cells from P1, implying less intracellular storage components (Fig. 6C and Fig. S4B). These observations indicated that cells from P2 may feature a reduced cultivability than cells from P1, which was confirmed by the clearly lower number of colony forming units (CFU) determined for live-sorted cells from P2 (Fig. 6D). To test a possible reversibility of such a cultivability difference, 9 × 10^5^ sorted, pelleted, and resuspended cells of both subpopulations were plated on BG11 agar plates and grown for 4 days (Streak 1 in Fig. 6E). The cells were transferred to fresh plates twice and incubated for additional 2 days (Streak 2) and 3 days (Streak 3). Remarkably, the reduced cultivability of P2 cells turned out to be reversible. They recovered upon continuous plating (Fig. 6E). Further, P1 and P2 originating from cultivation under HL-HC-LP conditions for 7 d (see also Fig. 4) were analyzed regarding GCN distributions. Interestingly, these subpopulations also differed in this regard, with P1 featuring C4n-C8n as prominently populated gates, whereas P2 showed a broader distribution with most cells in gates C8n, C9n, and Cxn (Fig. S5).

Overall, upon cultivation under LP conditions, *Synechocystis* cells were found to develop two subpopulations with clearly differing physiological characteristics, which, beside their size, included a differing shape, pigmentation, and cultivability. These subpopulations, constituting an interesting finding within this GCN study, deserve and are subject to further analyses regarding storage compound identities and content as well as differences regarding viability.

## Discussion

In the current study, we performed a comprehensive analysis of changes in the DNA content over time in *Synechocystis* sp. PCC 6803 cultivated over extended time ranges under different environmental conditions. Regardless of the growth condition, an increase of the GCN during exponential growth followed by an immediate drop upon growth arrest was observed. The dynamic changes of the GCN correlated well with the growth rate. During linear and stationary growth phases, the GCN was found to decrease to relatively low levels. Those findings verify the hypothesis that the GCN in *Synechocystis* is regulated in a growth-phase-dependent way as also postulated in earlier studies [5, 8, 17, 27]. This dependency on the growth phase also explains the strong divergence of reported absolute GCNs for *Synechocystis*, for which the exact growth state and conditions applied typically were not appropriately considered. We show that much higher GCN levels are reached than the typically reported 10–20. Further, the present study reports absolute GCNs determined by qPCR as well as GCN diversification between cells within a sample, as determined at the single-cell level by flow cytometry, all in a time-dependent manner. The extraordinarily high levels and dynamics of GCNs per cell in a population were found to strongly depend on the applied growth conditions and the growth phase. The highest GCN values and dynamics were observed for cultivation under HL-HC-HP conditions, a condition enabling unlimited growth for an extended time period. Any limitation applied (light, CO_2_, and/or phosphorus) resulted in lower GCN increases correlating with maximal growth at a lower rate and/or for a shorter time period. This verifies the close correlation between GCN and growth, which has been proposed in several studies, but not systematically demonstrated nor analyzed proportionally across a population [4, 13, 27–29]. It remains to be investigated, whether or to what extent the correlation between GCN and growth can be generalized for cyanobacteria and what specific effects limitations in phosphorus, CO_2_, and light have on population structure and physiology [13, 30].

Watanabe and coworkers demonstrated that cyanobacteria generally show an asynchronous type of cell division [27, 31]. Our study confirms this behavior, as we found an uneven distribution and also odd numbers of chromosomes in cells. Genome replication strategies however differ among different cyanobacteria [32]. Whereas genome replication in *S. elongatus* depends on the DNA replication initiation factor DnaA and *oriC*, *Synechocystis* features replication from multiple origins in a DnaA-independent manner [33]. Further, *S. elongatus* GCNs are limited ensuring a stable copy number per cell volume [30], corresponding with the cell size to protein content correlation [34], whereas *Synechocystis* strains feature highly variable GCNs. The cell size of *Synechocystis* was found in this study to increase with increasing GCN and growth rate as also reported before [27]. This size increase however did by far not reflect the increasing GCN.

The mean DAPI fluorescence of 37.3 [arb. FI units] in gate C1n and correspondingly higher fluorescence intensities in gates C2n-Cxn (Fig. 1), with all gates set according to Gaussian distributions considering all samples analyzed in the study, represent differing GCNs in cells of a population. As stated above, subpopulation C1n can be assumed to contain cells with one chromosome, which mainly manifested under phosphorus-limiting conditions. During exponential growth, most cells showed an exceptionally high DAPI fluorescence, especially under HL-HC-HP conditions, when some cells appeared to have extremely high GCNs of ~ 260 (DAPI fluorescence in the range of 10,000 [arb. FI units], Fig. S2). With 1 DNA copy (3.57 Mb equaling ~ 3.7 fg [35]), corresponding to about 0.03–0.07% of the total cell dry weight (5.3–11.3 pg per cell [36]), the 260 copies can be estimated to make up 7.7–18.2%. Moreover, under HL compared to LL conditions, the DNA content stayed higher during linear and stationary growth. In contrast to these observations, Zerulla et al. [8] reported a higher GCN (twice as high) during growth (OD = 0.1) under low light (30 µmol photons m^− 2^ s^− 1^) than under higher light (85 µmol photons m^− 2^ s^− 1^) conditions. The applied light intensities were however lower than those applied in this study (50 and 200 µmol photons m^− 2^ s^− 1^), and cell growth limitations may have been different. High GCN levels may generally compensate increased oxidative stress at high light intensities [14], conditions promoting DNA damage and leading to a high demand for repair processes and metabolic energy [28]. Thereby, the high GCN can be proposed to fulfill two roles, maintaining a stock of intact DNA and serving as an energy storage.

Gärtner and co-workers observed more than three-fold higher GCNs during exponential growth at 2% CO_2_ compared to ambient CO_2_ [15]. Time courses have however not been analyzed and the GCN for cultivation at ambient CO_2_ was very low. The current study shows that, under LL conditions, CO_2_ only has a minor effect on GCN dynamics and the average GCN in subpopulation Cxn reached up to 19 under LL-HC-HP conditions, whereas CO_2_ availability had a strong effect under HL conditions, with the average GCN in subpopulation Cxn reaching up to 72 under HL-HC-HP conditions.

From a biotechnological perspective, GCN dynamics are of high interest because genome replication may constitute a possible metabolic burden [37] and because high GCNs may hamper genetic modifications on the genomic level as full segregation is necessary but often difficult to achieve. In this context, it is interesting to explore conditions, under which cells maintain only one chromosome. Whereas all limitations tested in this study led to strong GCN reduction, the latter was clearly most pronounced under phosphorus limitation with most cells accumulating in the C1n and C2n subpopulations at the end of the stationary phase (Fig. 4). In agreement with this observation, Zerulla and co-workers reported a monoploid status after extended cultivation in total absence of phosphate [8]. This monoploid status is supported by our study although only a fraction of cells (C1n) reached this state. Cultures in this study were grown with low phosphate concentrations (i.e. 17.5 µM) and not in complete absence of phosphate, which may further increase the occurrence of monoploid cells. Under non-limiting phosphorus conditions, light was the next most pronounced factor influencing the GCN. LL-LC-LP conditions led to the lowest GCN levels indicating an additive effect of the different limitations.

For phototrophic organisms, Rees and Raven revealed a direct correlation of cellular phosphorus content in various compounds like DNA and RNA with the growth rate [38]. In this study, we consistently observed initially unrestricted growth involving a strong GCN increase until a limitation occurred, e.g., by phosphorus. This highlights the priority of DNA synthesis during the first hours of cultivation, for which phosphorus assimilation in the lag phase, as reported for *S. elongatus* [39], may constitute a preconditioning. Under the conditions applied in this study, explicit lag phases were not observed for *Synechocystis*, which has been reported to store and mobilize phosphorus via polyphosphate and nucleic acid polymers such as rRNA [40–42]. The GCN dynamics observed in this study under low phosphate conditions, including lower GCN maxima and minima, imply a role of the GCN in phosphate storage as postulated before [13, 43]. Phosphorus depletion after initial growth led to cells with exceptionally low GCNs. A storage function of high GCNs could contribute to the “luxury P uptake” strategy of cyanobacteria, i.e., the ability to take up more P than necessary for immediate growth, a strategy found in *Synechocystis* under unlimited conditions [40, 44–46].

To our knowledge, the occurrence of two subpopulations of differing cell size upon phosphorus limitation has not been reported before for cyanobacteria. These subpopulations were found to differ in cell shape, storage compound content, pigment content, and growth capabilities. Cyanobacteria are known to withstand various stresses [47, 48] and thereby may develop cells with differing physiological state to be prepared for environmental changes. Under LP conditions, a fraction of cells may invest in storage compound synthesis (P1, Fig. 6C) facilitating survival after phosphate depletion, while the other fraction continues to proliferate, ending up in strong starvation upon phosphate depletion, and then has to rely on DNA, pigments, and the cell membrane as phosphorus and energy sources (P2). As a consequence, cell size and membrane properties may change affecting cell division and viability.

Changes in autofluorescence have been reported as a reaction to other environmental conditions, such as nutrient limitations leading to chlorosis, i.e., the loss of photopigments and switch to a resting cell state [16, 49]. In *Prochlorococcus*, this metabolic adaption was described to involve the formation of subpopulations differing in chlorophyll autofluorescence [50]. Cellular autofluorescence heterogeneity has also been observed during the recovery from prolonged iron deprivation [51]. In this case, only cells with high autofluorescence were able to grow and reconstitute thylakoid membranes. The P2 cells of the small-cell-sized subpopulation were less cultivable, yet they survived and recovered after being transferred to P-rich conditions (Fig. 6E), highlighting *Synechocystis’s* remarkable survival strategies. Anyway, more research is required to get insight into this behavior, for which the observed subpopulations may serve as a highly promising starting point.

## Conclusions

GCN dynamics in *Synechocystis* were found to strongly depend on the growth stage with a very pronounced GCN increase during unlimited growth when average GCNs of > 70 were reached and subsets of cells showed GCNs of well above 100. The on-set of a limitation, especially of phosphorus, led to a strong GCN decrease and the establishment of subpopulations with only a single chromosome in late strongly limited growth stages. GCN dynamics under P-limited conditions highlight the role of the GCN in phosphate storage. Besides phosphorus, CO_2_, and light were found to be factors influencing the GCN. Under LL conditions, the effect of CO_2_ on the GCN dynamics was minor, whereas CO_2_ becomes a dominating factor under HL conditions. The highest GCN dynamics in the course of growth progression were observed under unlimited conditions (HL-HC-HP). These findings give novel insights on GCN dynamics and respective influencing factors in *Synechocystis* and resolve divergences in literature regarding maximum GCNs and GCN dynamics. Generally, elucidating GCN distributions can be used to determine the growth state of cells and is relevant for strain engineering (transformation, pathway engineering, etc.), i.e., for GCN minimization for genome modification. Further, courses of GCN distributions can give novel insights into physiological adaptations of cyanobacteria in response to environmental variations and inform cultivation strategies in engineering contexts. Besides, this work revealed the development of subpopulations that differ in terms of their physiological characteristics (i.e., cell size, cell shape, pigmentation, storage components, and cultivability) upon phosphorus starvation, which deserves further investigation. Such cell heterogeneity may be a means of adapting to demanding environmental conditions, thus highlighting *Synechocystis’s* remarkable survival strategies.

## Supplementary Information

Below is the link to the electronic supplementary material.

Supplementary Material 1.

## Data Availability

All data generated or analyzed during this study are included in this published article and its supplementary information files or provided at public repositories. Cytometric raw data can be accessed at Zenodo ( https://zenodo.org/records/17241600? ). Further data (OD 750 , growth rates, qPCR data and microscope pictures) also are available in the Zenodo repository, https://doi.org/10.5281/zenodo.16778049. Additional information is available from the corresponding author on reasonable request.

## Abbreviations

CFU: Colony forming units
Cn1-Cnx: Gates defined in 2D dot plots of flow cytometric analyses, corresponding to subpopulations containing a specific GCN (Cn1-Cn9) or higher GCNs (Cnx)
DAPI: 4’,6-diamidino-2-phenylindol
FI: Fluorescence intensity
FSC: Forward scatter
GCN: Genome copy number
gDNA: Genomic DNA
HC: High CO_2_ level (2%)
HIM: Helium ion microscopy
HL: High light (200 µmol photons m^− 2^ s^− 1^)
HMDS: Hexamethyldisilazane
HP: High phosphate concentration (175 µM)
LC: Low CO_2_ level (ambient, 0.04%)
LL: Low light (50 µmol photons m^− 2^ s^− 1^)
LP: Low phosphate concentration (17.5 µM)
P1 and P2: Subpopulations emerging under phosphate starvation
PBS: Phosphate buffered saline
PFA: Paraformaldehyde
RT: Room temperature
SDEV: Standard deviation
SSC: Side scatter
STEM: Scanning electron microscopy in transmission mode
*Synechocystis*: *Synechocystis *sp. PCC 6803
qPCR: Quantitative PCR
