# Supplementary material for: Synechocystis sp. PCC 6803 shows high cell cycle dynamics reflected by an extraordinary genome copy number variation
Source: Microb Cell Fact. 2026 Apr 27;25:113. doi: 10.1186/s12934-026-02982-3 (PMC13126801; doi:10.1186/s12934-026-02982-3)
Supplement: Supplementary file 1 — Supplementary Material 1. [file 12934_2026_2982_MOESM1_ESM.docx]

*Synechocystis* sp. PCC 6803 shows high cell cycle dynamics reflected by an extraordinary genome copy number variation

Authors: Till, Jacky^1,2^; López Gálvez, Juan^3^; Schattenberg, Florian^3^; Schmidt, Matthias^1,4^; Müller, Susann^3^; Toepel, Jörg^1*^; Bühler, Bruno^1,5^

Affiliation: ^1^ Department of Microbial Biotechnology, Helmholtz Centre for Environmental Research – UFZ, Permoserstr. 15, 04318 Leipzig, Germany. ^2^ Institute of Chemical, Environmental and Bioscience Engineering, TU Wien, Gumpendorfer Str. 1a, A-1060 Vienna, Austria. ^3^ Department of Applied Microbial Ecology, Helmholtz Centre for Environmental Research – UFZ, Permoserstr. 15, 04318 Leipzig, Germany. ^4^ Department of Technical Biogeochemistry, Helmholtz Centre for Environmental Research – UFZ, Permoserstr. 15, 04318 Leipzig, Germany. ^5^ Faculty of Natural Sciences I – Biosciences, Martin-Luther-University Halle-Wittenberg, Kurt-Mothes-Str. 3, 06120 Halle (Saale), Germany.

^*^ Corresponding author: [joerg.toepel@ufz.de](mailto:joerg.toepel@ufz.de)

Supplementary Information


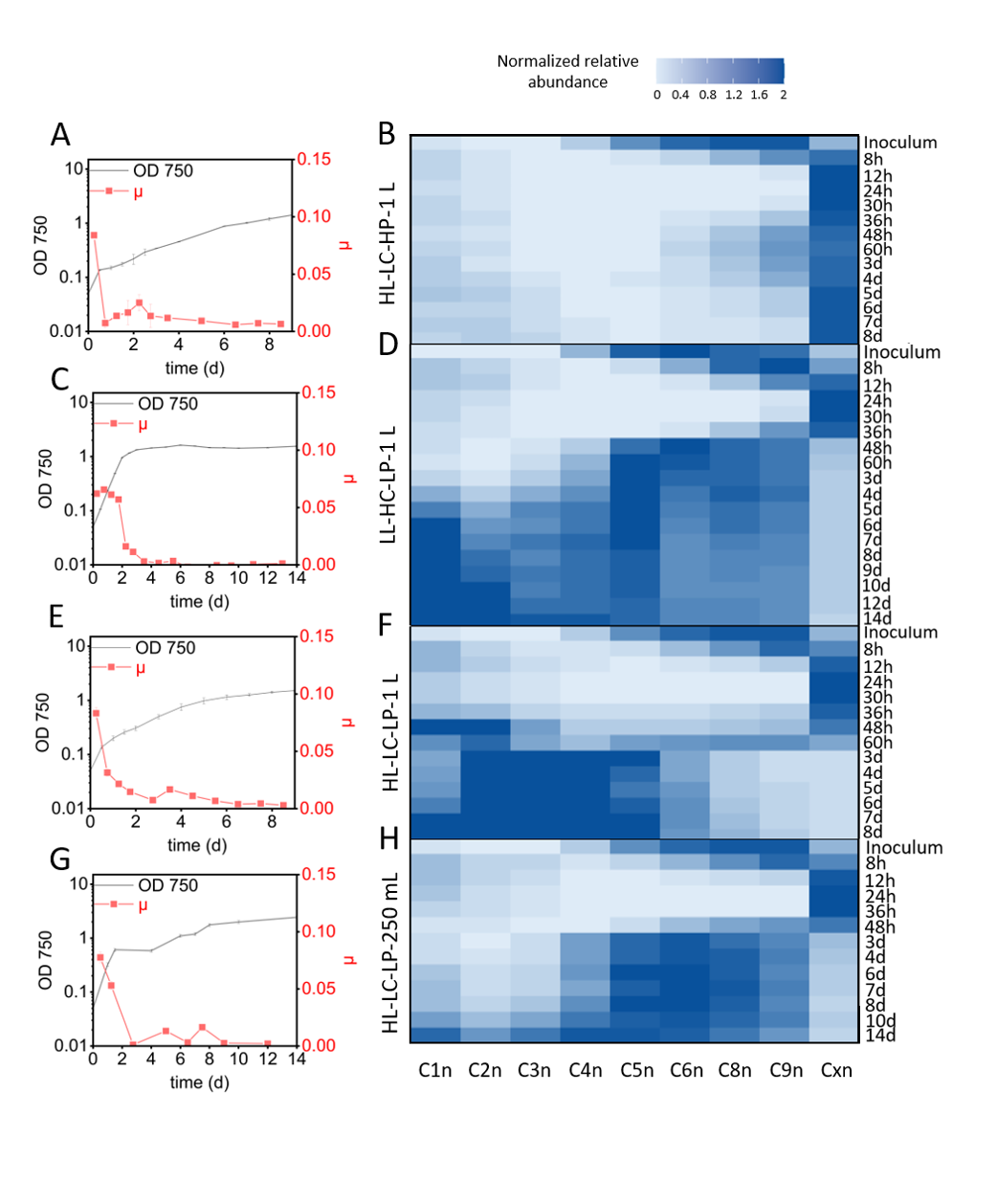


Figure S1: Growth- and time-dependent GCN dynamics in *Synechocystis* cultivated under various conditions. Cultures grown at HL-LC-HP (panels A and B), LL-HC-LP (panels C and D), and HL-LC-LP (panels E and F) conditions in 1 L flasks and under HL-LC-LP conditions in 250 mL flasks (panels G and H) were analyzed. Growth curves (OD_750_) are plotted in panels A, C, E and G for all conditions, together with µ courses based on values calculated between sampling time points. Panels B, D, F and H show GCN patterns over time analyzed by flow cytometry on the basis of DAPI fluorescence, with the cell signals assigned to defined gates (see Fig. 1 and Materials and Methods for experimental details). Darker colors represent higher relative cell numbers of cells per gate.


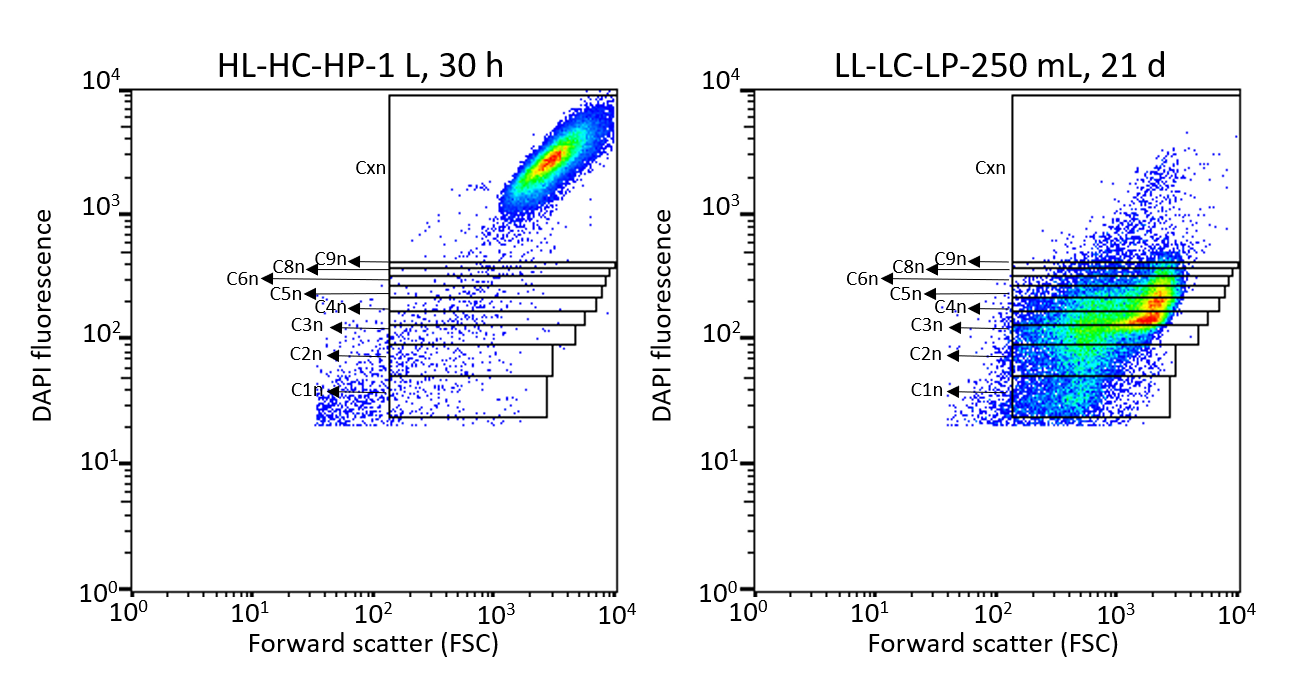


Figure S2: 2D dot plots of FSC vs. DAPI fluorescence including the gate template. Left: Fluorescence intensities shown for cells grown for 30 h under HL-HC-HP conditions in 1 L flasks. Right: Fluorescence intensities shown for cells grown for 21 d under LL-LC-LP conditions in 250 mL flasks.


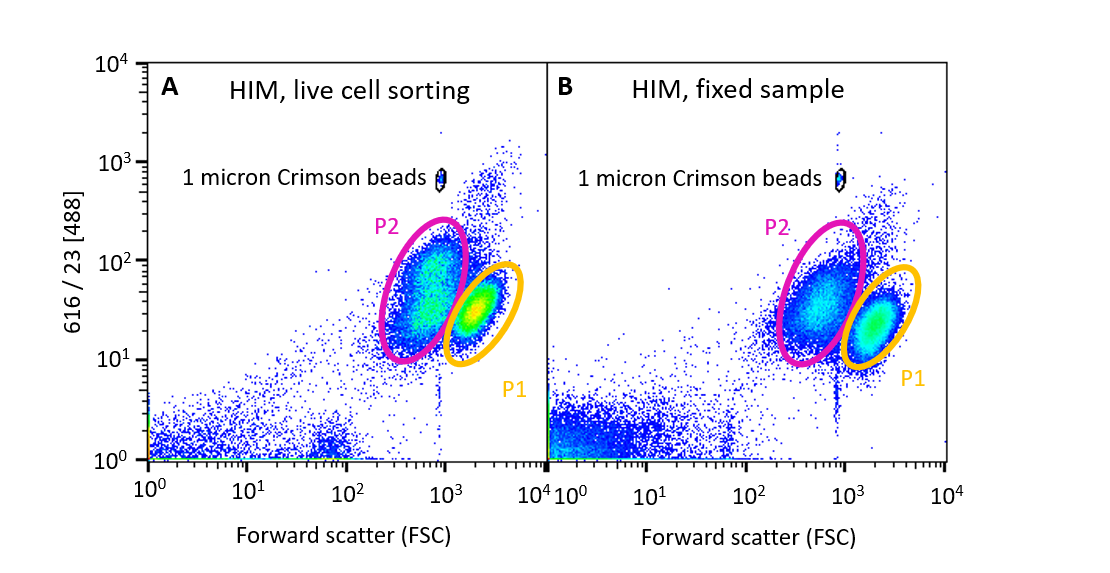


Figure S3: *Synechocystis* subpopulations with different cell sizes formed after cultivation under HL-HC-LP conditions for 7 d. Panel **A** shows a 2D dot plot of FSC vs. autofluorescence at 616 nm +/- 23 nm upon excitation at 488 nm for living cells and the formation of subpopulations P1 and P2, which were live-cell-sorted by flow cytometry for HIM and STEM analysis (see Fig. 6). Panel **B** shows the control for the same cells after fixation with 2% PFA and DAPI staining as described in section 2.5, in order to demonstrate that fixation does not significantly alter the cell morphology. It shows the same pattern as samples taken after 7 d during the HL-HC-LP-1 L experiment described in section 3.4 (see Figure 6A).


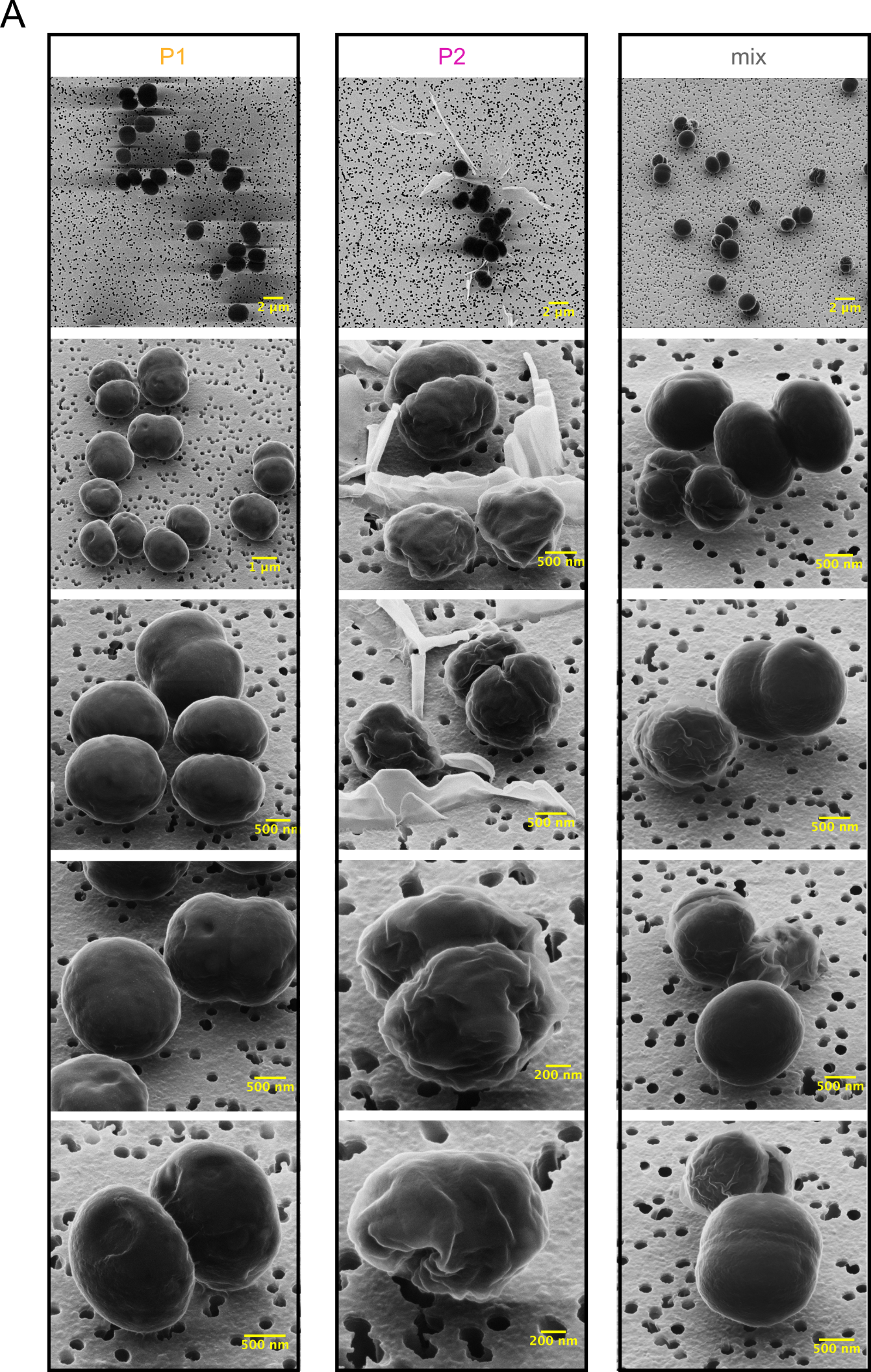


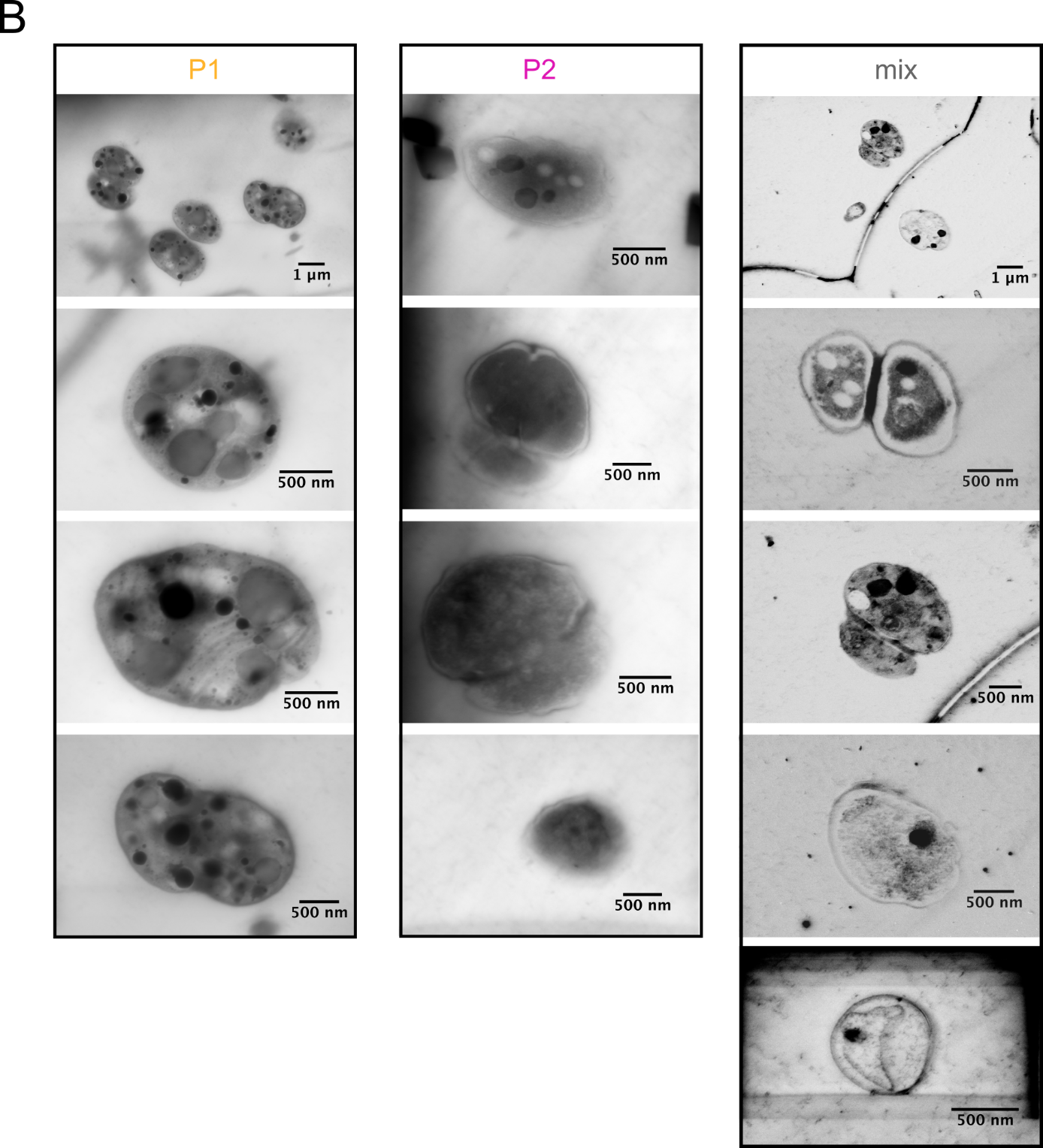


Figure S4: Microscopy analysis of *Synechocystis* subpopulations formed after growth under HL-HC-LP conditions for 7 d. Subpopulations P1 and P2 obtained from live-cell-sorting and unsorted cells (mix) were analyzed via HIM (A) and STEM (B).





Figure S5: Flow-cytometry-based GCN analyses of *Synechocystis* cells in subpopulations P1 and P2 differing in cell sizes (FSC, forward scatter) and formed after cultivation under HL-HC-LP conditions for 7 d. The two subpopulations P1 and P2 separated in the FSC vs. autofluorescence (616 nm +/- 23 nm, Figure 6a) are visualized singly in FSC vs. DAPI fluorescence 2D-plots to analyze GCNs separately. Each dot in the 2D plots represents a cell, and the color changes from blue to red refer to increasing numbers of cells per position. The overlay represents the gate template applied to count proportions of cells in subpopulations according to their DAPI fluorescence intensity with the gates C1n for 1 GCN per cell to Cxn for more than 9 GCNs per cell (see also Fig. 1).
